# MAXIMIZING COGNITIVE TRAINING OUTCOMES WITH SIMULTANEOUS BRAIN STIMULATION

**DOI:** 10.1093/geroni/igae098.2189

**Published:** 2024-12-31

**Authors:** Susanne Jaeggi

**Affiliations:** Northeastern University, Boston, Massachusetts, United States

## Abstract

Cognitive health is a fundamental issue facing everyone across their lifespan, however, while there is accumulating evidence for the efficacy of targeted interventions to improve cognition, the outcomes have been inconsistent. This contrasts the vast basic science literature suggesting that brain circuits are highly malleable, even in older age. Non-invasive transcranial direct current stimulation (tDCS) has shown to facilitate brain plasticity, especially when administered during a learning event. Here, I will summarize the results of several randomized controlled trials as well as a meta-analysis conducted by my group focusing on cognitive training that is paired with tDCS. Across 4 separate studies involving 257 younger and older adults, we found evidence for improved learning trajectories as a function of tDCS, and in addition, we observed improvements in measures of long-term memory, some of which remained significant several months after training completion. The effects on working memory outcomes appear to be more mixed and dependent on baseline ability, i.e., those with initial lower performance typically benefit more from brain stimulation. Our primary findings are supported meta-analytically in that we found a small positive net effect of tDCS on transfer measures after working memory training, both, in the short- and long-term. However, the effects were most pronounced for measures that were similar to the trained tasks. Collectively, our studies illustrate the potential of tDCS to support cognitive training outcomes, but at the same time, there are boundary conditions and important individual differences that moderate training efficacy.

